# Physical pressure resistance of gastrointestinal anastomotic site via plate of polyglycolic acid promoting fibrosis

**DOI:** 10.1038/s41598-024-77894-6

**Published:** 2024-10-30

**Authors:** Hideki Tanda, Masatsune Shibutani, Seiji Natsuki, Hiroaki Kasashima, Tatsunari Fukuoka, Kiyoshi Maeda

**Affiliations:** Department of Gastroenterological Surgery, Osaka Metropolitan University Graduate School of Medicine, 1-4-3 Asahi-machi Abeno-ku, Osaka City, Osaka Prefecture 545-8585 Japan

**Keywords:** Polyglycolic acid, Anastomotic leakage, Animal experiment, Physical pressure resistance, Colorectal cancer, Techniques and instrumentation

## Abstract

Anastomotic-leakage incidence has been reported to be reduced on using polyglycolic acid (PGA) sheets as reinforcing materials; however, there is insufficient evidence regarding the reinforcement mechanism. Therefore, we investigated effects of PGA sheets on gastrointestinal anastomoses in rats. In the first approach, five rats underwent laparotomy; the PGA sheet was pasted onto the normal cecal wall. After five days, the cecum was removed and histologically evaluated. In the second approach, ten rats were randomly divided into two groups of five animals each. The rats underwent laparotomy; the cecal wall was sutured after a full-thickness incision. In the PGA group, a PGA sheet was used to cover the suture area. After 5 days, the cecum was removed, and the physical pressure resistance was evaluated. We confirmed the growth of a fibrous capsule measuring 855 (648–1048) µm outside the cecal serosa in the first approach. The median pressure resistance in the second approach was 57.0 (45.0–90.0) and 90.0 (82.5–94.5) mmHg in Control and PGA groups, respectively. The pressure resistance was significantly higher in the PGA group (*p* = 0.046). In summary, the PGA sheet may form a barrier of fibrosis on the intestinal wall and provide reinforcement to prevent anastomotic leakage.

## Introduction

Anastomotic leakage (AL) is a postoperative complication of great concern following gastrointestinal surgery. In patients undergoing colorectal cancer surgery, AL is associated with serious issues such as peritonitis and sepsis and is linked to increased mortality^1^. AL is also associated with longer hospital stay, increased medical expenses^2^, and negatively affects long-term survival^3^. According to the Japanese National Clinical Database (NCD), the incidence of AL is currently reported at approximately 10%^4^. Several surgical techniques have been developed for this purpose. For instance, indocyanine green is used to evaluate intestinal blood flow^5^. The placement of a transanal tube is also used for decompression of an anastomosis^6^. However, its incidence is reportedly > 5%. Although adding sutures to reinforce the anastomosis^7^ in both laparoscopic and open surgery is useful, it is often difficult to apply reinforcing sutures, particularly in patients treated with very low anterior resection, because of the limited, narrow pelvic field.

Polyglycolic acid (PGA) is not enzymatically degraded but is hydrolyzed. PGA sheets are effective in preventing air leakage and pancreatic fistulas after lung and pancreatic surgery, respectively^8,9^. In a previous study by our department, we reported that reinforcing rectal anastomosis using PGA sheets reduced the incidence of AL^10^. PGA sheets may also be useful in gastrointestinal anastomoses. However, the causes of AL are diverse, including the presence or absence of comorbidities and intraoperative complications^11^. Therefore, it is difficult to confirm whether the incidence of AL is reduced by PGA using actual clinical data. Therefore, this study aimed to investigate the effect of PGA sheets in intestinal anastomosis by verifying changes in the microscopic tissues over time and measuring pressure resistance in experimental animals.

## Methods

### Animals

Female Sprague-Dawley (SD) rats (6–8 weeks old; Clea Japan, Tokyo, Japan) were used. All animal experiments were performed in accordance with the Guide for Animal Experimentation of Osaka Metropolitan University and ARRIVE guidelines. All efforts were made to minimize suffering. The study protocol was approved by the ethics committee of Osaka Metropolitan University (approval number: 23037).

### Experimental procedure 1–1 (attaching the PGA sheet to the normal cecal wall)

Three rats were used. Rats were administered isoflurane via inhalation for anesthesia. The isoflurane concentration was maintained at 2–3%. Following the induction of anesthesia, a 2-cm midline abdominal incision was created. The cecum was identified and a PGA sheet (NEOVEIL^®^; Gunze, Kyoto, Japan) was applied to the serosal surface contralateral to the mesentery. The PGA sheet was trimmed to a size of 1 cm × 3 cm, and the two sheets were superimposed. Two sutures were created using 6-0 Nylon suture (Nescosuture^®^, Alfresa Pharma, Japan) thread to secure the cecum and PGA sheet. The abdominal wall was closed with two layers of 4-0 polysorb (Covidien Japan, Tokyo, Japan). The rats were euthanized by cervical dislocation after 3, 5, or 7 d. To observe the tissue changes over time, each cecum was removed and specimens were prepared.

### Experimental procedure 1–2 (tissue evaluation after 5 days)

Five rats were used in this study. PGA sheets were applied to the cecum of the rats using the procedure described above. All rats were sacrificed after 5 days, the cecum was removed, and specimens were prepared.

### Experimental procedure 1–3 (specimen preparation)

Specimens were cut in the short axis direction, fixed in 10% neutral buffered formalin solution for 24–48 h, embedded in paraffin, and sliced to a thickness of 4 μm. Hematoxylin and eosin (H&E) and Masson’s trichrome staining (MTS) were performed. MTS was performed using a Trichrome Stain Kit (ScyTek Laboratories, USA). All procedures were conducted according to the manufacturer’s instructions. All sections were deparaffined, rehydrated, and then washed in distilled water, re-fixed in Bouin’s Fluid for 60 min, rinsed with running water, rinsed with distilled water, stained with Weigert’s iron hematoxylin solution for 2–4 min, rinsed with running tap water for 2 min, rinsed with distilled water, stained with Biebrich scarlet solution for 5–10 min, rinsed with distilled water, differentiated in phosphomolybdic-phosphotungstic acid solution for 10 min, aniline blue was applied for 5–10 min. The specimens were then rinsed with distilled water, acetic acid solution (1%) was applied for 3–5 min, dehydrated, cleared with xylene, and mounted with Marinol (Muto Chemical Co., Ltd., Tokyo, Japan).

### Experimental procedure 1–4 (histological evaluation)

Stained slides were captured with Aperio ScanScopeCS2^®^ (LeicaMicrosystems, Wetzlar, Germany) and evaluated using QuPath software version 0.5.1 (https://qupath.github.io), which is an open-source digital pathology tool^12^. For the rat specimens prepared as described in experimental procedure 1–2, the thickness of the normal cecal wall and that of the capsule containing extraserous fibrotic areas (collagen fibers), which were stained blue with MTS, were measured. For the normal intestinal walls, the distance from the mucosal surface layer to the serosa was measured in the vertical direction. For extraserous fibrotic areas, the distance from the cecal serosa surface to an area with dense cell infiltration and fibrosis was measured vertically. Measurements were performed at five locations for each sample, and the median value was used for evaluation.

### Experimental procedure 2–1 (attaching the PGA sheet to the sutured cecal wall)

**Fig. 1:**
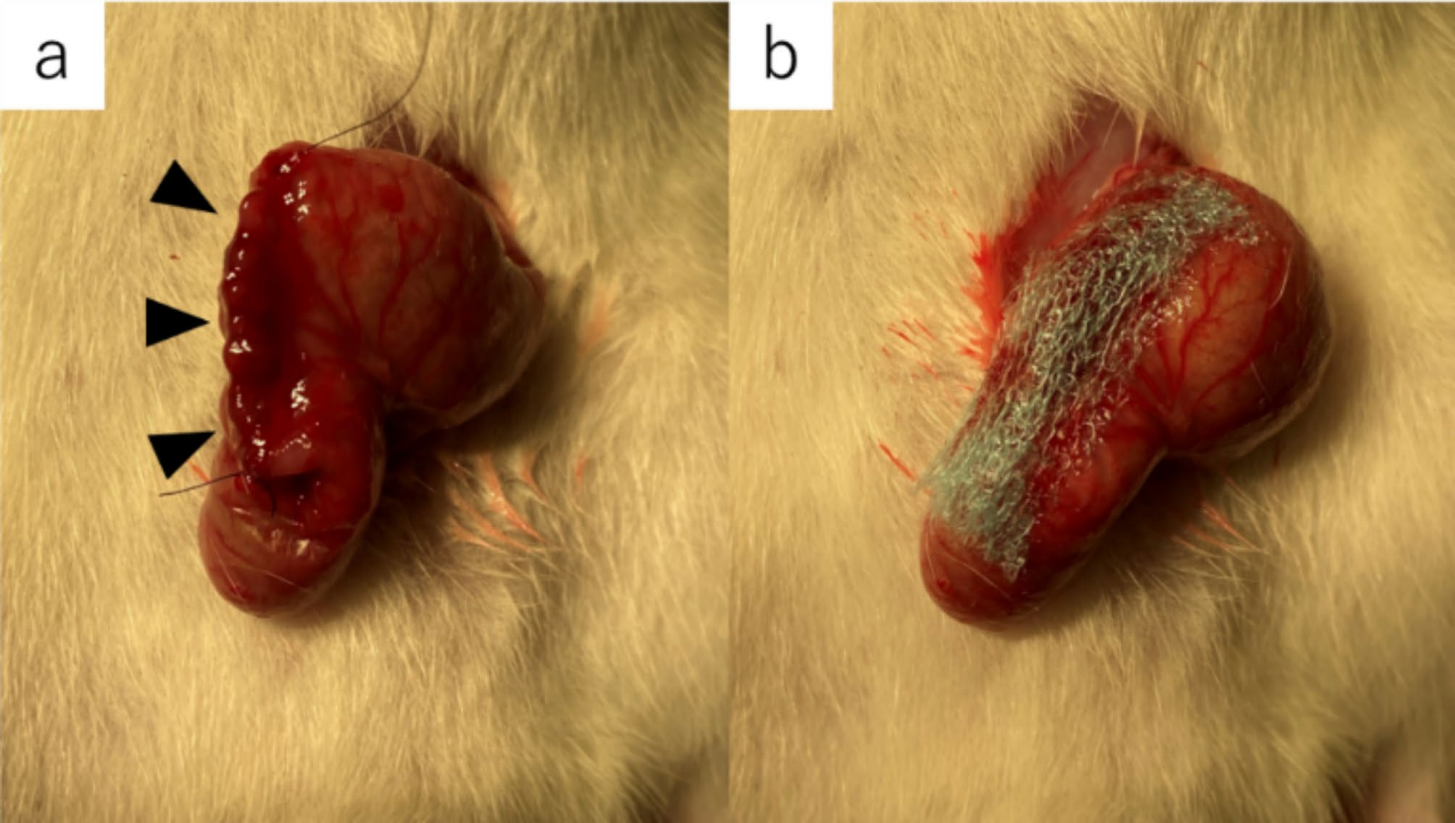
Suturing the cecal wall and applying PGA. (**a**) Suture area (black arrowheads). (**b**) PGA sheet attached to the suture area. *PGA* polyglycolic acid.

Ten rats were randomly divided into control and PGA groups of five rats each. Similar to experimental procedure 1–1, inhalation anesthesia was performed and a 2-cm midline incision was created in the rat and the cecum was identified. A 1-cm full-thickness incision was created in the cecal wall contralateral to the mesentery. To prevent contamination, feces in the cecum were collected and discarded as much as possible. The incised cecal wall was closed in one layer with a full-thickness continuous suture using 6-0 Nylon suture. For the five animals in the PGA group, a double-layer PGA sheet trimmed to 1 cm × 3 cm was attached to the suture site and fixed using two 6-0 Nylon sutures (Fig. 1).

In the Control group, the PGA sheet was not applied and only suture closure was performed. In both the Control and PGA groups, the abdominal wall was closed with two layers of 4-0 polysorb.

### Experimental procedure 2–2 (pressure test)

**Fig. 2:**
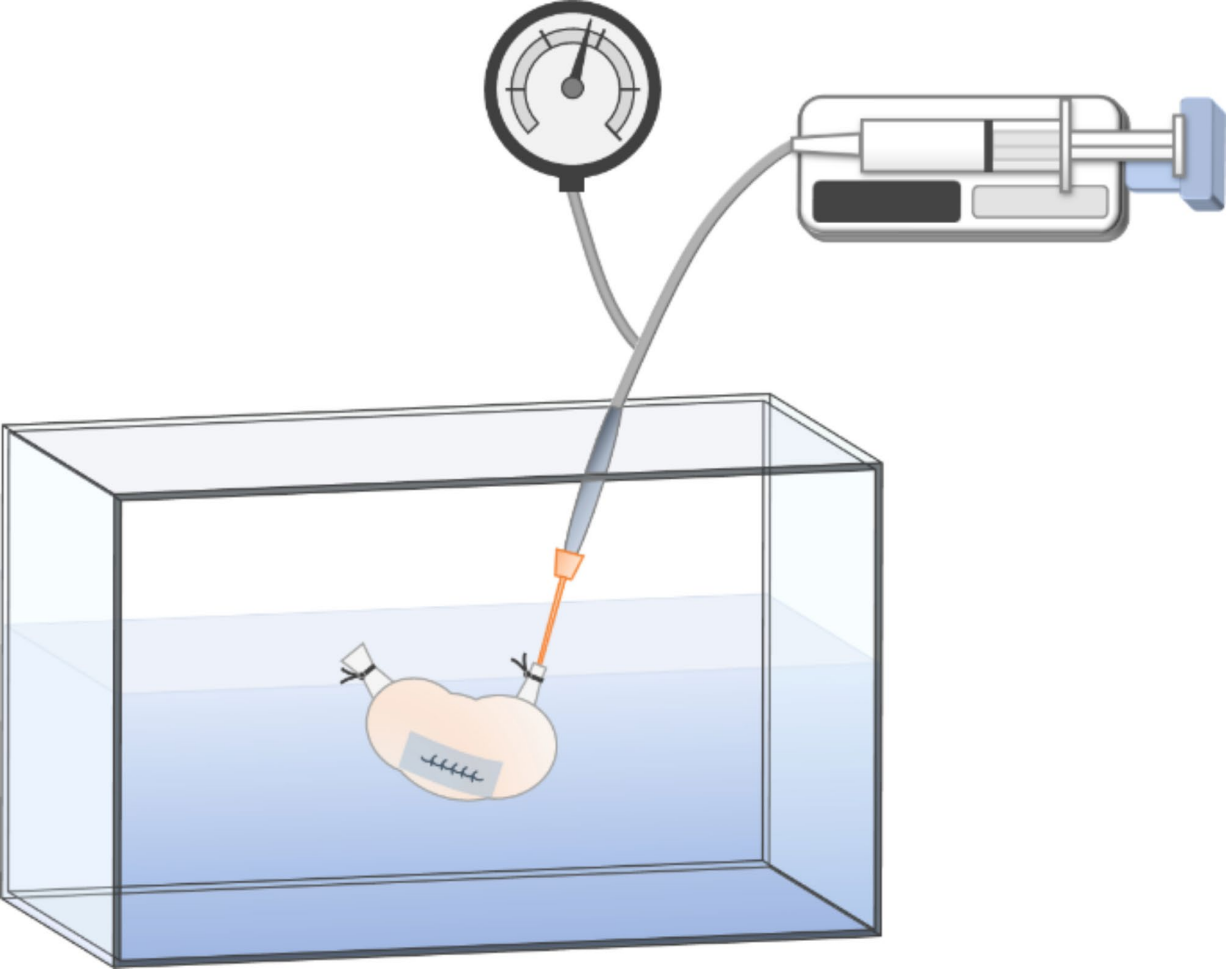
Schema of the pressure test.

The rats were sacrificed after 5 days. The cecum and at least 2 cm of the ileum on the oral side and the colon on the anal side were removed. The colonic stump was ligated using a 3-0 nylon thread. The intravenous catheter of a 20G indwelling needle (Surflow^®^, Terumo, Tokyo, Japan) was inserted through the stump on the ileum side, and the catheter and ileum were ligated and fixed using 3-0 nylon thread. A 50 mL syringe filled with air was installed in a syringe pump, and a micromanometer (MIGISHITA SEIKI MFG. Co., Ltd, Hyogo, Japan) were connected in the “Y” configuration. They were connected to a catheter (Fig. 2).

Air was injected into the segment at a rate of 2 mL/min while the segment was submerged in water. The maximum pressure recorded until the air bubbles were confirmed at the suture site was recorded as the bursting pressure.

### Statistical analysis

Statistical analyses were performed using the EZR statistical software version 1.55 (Saitama Medical Center, Jichi Medical University, Saitama, Japan) and a graphical user interface for R version 1.61 (R Foundation for Statistical Computing, Vienna, Austria). The EZR statistical software is a modified version of the R commander designed to add statistical functions frequently used in biostatistics. The bursting pressures were compared using the Mann–Whitney U test. Statistical significance was set at *p* < 0.05. A graph of the pressure test results was created using the GraphPad Prism software version 10.0 (La Jolla, CA, USA, https://www.graphpad.com/).

## Results

### Tissue changes over time

**Fig. 3:**
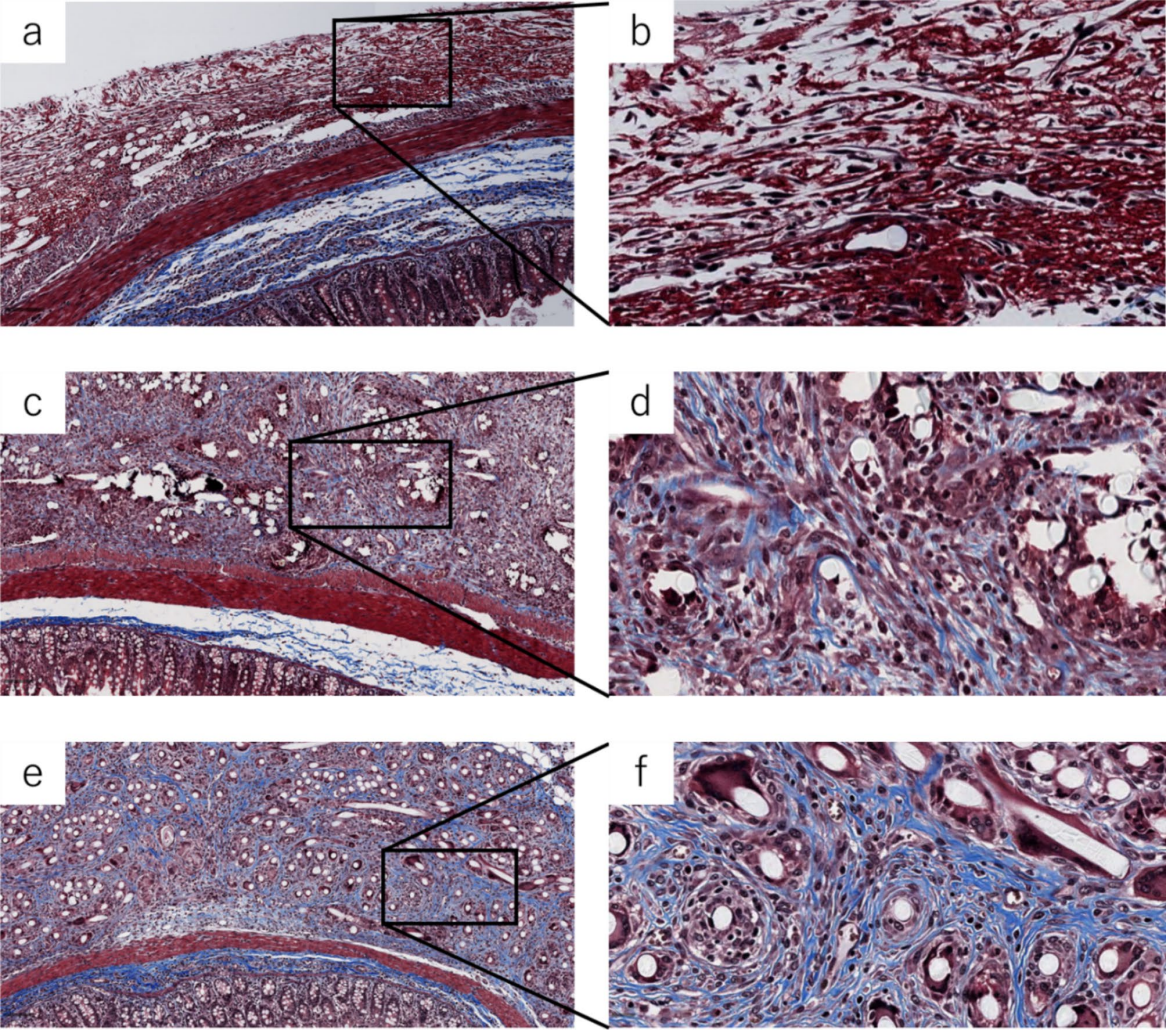
Histological findings of PGA sheet attached to normal cecal wall. (**a**) Masson Trichrome Staining (MTS) on Day 3. Low magnification (scale bar, 100 μm). (**b**) MTS, Day 3. High magnification; scale bar, 20 μm. (**c**) MTS, Day 5. Low magnification; scale bar, 100 μm. (**d**) MTS, Day 5. High magnification; scale bar, 20 μm. (**e**) MTS, Day 7. Low magnification; scale bar, 100 μm. (**f**) MTS, Day 7. High magnification; scale bar, 20 μm. *PGA* polyglycolic acid.

From a relatively early stage after surgery, infiltration of inflammatory cells was observed within the fibers of the PGA sheet in the MTS specimens at 3, 5, and 7 days. On the 3rd day, almost no blue-stained fibrotic areas were observed; however, on days 5 and 7, increased fibrosis was observed. The extraserous fibrotic area, stained blue, tended to increase over time after 3, 5, and 7 days (Fig. 3).

### Growth of fibrous capsule

The results of the thickness of the normal cecal wall and that of extraserous fibrotic areas are presented in Table 1.

**Table 1:** Thickness measurement result.

	Normal intestinal wall (*n* = 5)	Fibrotic area (*n* = 5)
Median of thickness (µm)	603	855
(Range)	(549–622)	(648–1048)

**Fig. 4:**
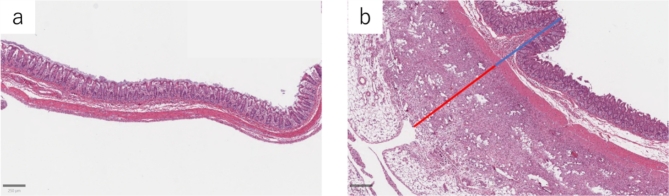
Measurement of fibrous capsule. (**a**) Normal cecal wall; scale bar, 250 μm. (**b**) Measurement of the distance from the mucosa to the serosa (blue line) and the area with dense fibrous membrane outside the serosa (red line); scale bar, 250 μm.

The median thickness of the five specimens was 603 (549–622) µm for the normal intestinal wall, whereas the median thickness of the extraserous fibrotic area was 855 (648–1048) µm (Fig. 4).

### Bursting pressure results

The results of pressure test were showed in Table 2.

**Table 2:** Results of bursting pressure.

	Control group (*n* = 5)	PGA group (*n* = 5)	*P* value
Median bursting pressure (mmHg)	57.0	90.0	0.046
(Range)	(45.0–90.0)	(82.5–94.5)	

*PGA* polyglycolic acid.

**Fig. 5:**
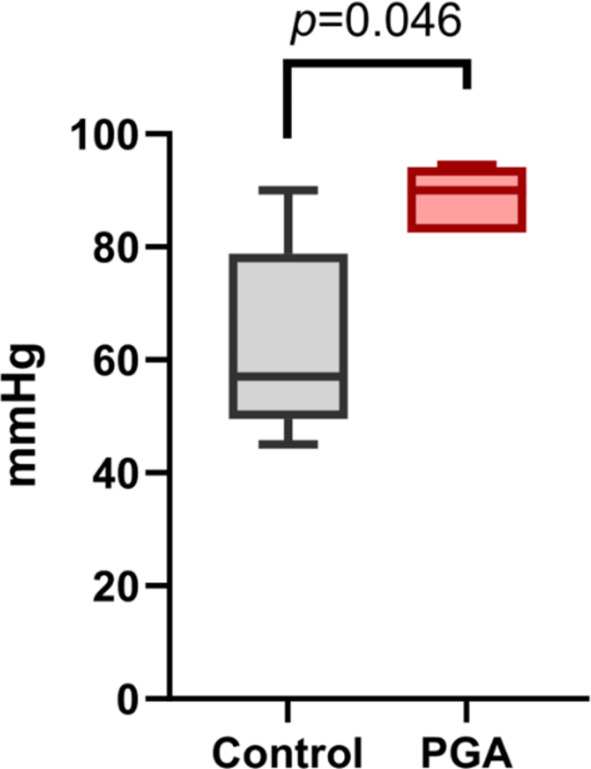
Bursting pressure. *PGA* polyglycolic acid.

The median bursting pressure values were 57.0 (45.0–90.0) mmHg in the Control group and 90.0 (82.5–94.5) mmHg in the PGA group. Hence, the PGA group exhibited a significantly higher pressure resistance (*p* = 0.046) (Fig. 5).

## Discussion

We observed that PGA sheets applied to the intestinal serosa caused fibrous capsules to grow over time. Physical pressure resistance improved in the PGA group, suggesting that the intestinal wall was reinforced via fibrosis promoted by PGA.

The strength of the intestinal wall is primarily derived from the collagen fibers located in the submucosa, which comprise the intestinal skeleton. Several days after surgery, the collagen levels at the anastomotic site decrease, thereby increasing the risk of AL. However, the strength of the anastomotic site is restored by newly formed collagen fibers^13^. Therefore, the growth of collagen is essential for anastomotic healing. Therefore, collagen fiber proliferation promoted by PGA may provide increased anastomotic strength.

PGA is non-enzymatically hydrolyzed to produce glycolic and lactic acids, triggering inflammation due to acidification, and is subsequently metabolized to produce water and carbon dioxide within 15 weeks^9^. PGA sheets can play an important role as scaffolds, inducing the infiltration of inflammatory cells in the early postoperative period. This results in the recruitment of fibroblasts, which form a physical barrier of fibrosis and ultimately serve as a scaffold to promote the formation of granulation tissue, which is required to promote tissue regeneration^14,15^. The PGA sheet has been widely applied in surgeries involving various organs to reduce postoperative complications^8,9,16^. Takagi et al. showed that this scaffold reduced the rate of postoperative pancreatic fistulas^8^. Early closure of the pancreatic leakage due to granulation formation may have been achieved, and the barrier formed by the infiltration of abundant fibroblasts within the PGA sheet scaffold may have prevented postoperative pancreatic fluid fistulas.

We previously reported the usefulness of double-stapling technique anastomosis combined with PGA sheets for rectal cancer surgery. Although a small number of cases were evaluated in this report, we observed that the incidence of anastomotic leakage in the PGA sheet group was significantly lower even after propensity score matching^10^. Choi et al. reported a case in which AL after total gastrectomy was cured by the oral application of a PGA sheet to the leakage site via an endoscope^15^. Hasegawa et al. reinforced the anastomosis with a PGA sheet in the small intestine of pigs and observed an increase in physical pressure resistance immediately after surgery^17^. Therefore, the usefulness of PGA has been confirmed, including the data from animal experiments. However, there are no reports of sequential evaluation of tissue or physical pressure resistance several days after surgery; therefore, Therefore, the present study might fill a gap in the literature.

As mentioned above, PGA promotes fibrosis during hydrolysis and is thought to be useful in preventing pulmonary air and pancreatic fluid leakage. We observed increased fibrosis in the intestinal walls. To verify whether PGA actually played a reinforcing role, we conducted a pressure resistance test and found that physical pressure resistance was significantly higher in the PGA group (*p* = 0.046). These results support the hypothesis that the PGA sheet forms a physical barrier owing to increased fibrosis and plays a role in reinforcing the anastomosis. In clinical practice, AL often occurs within 7 days after surgery^18^. The strength of the anastomosis decreases significantly within 3–4 days after surgery due to structural changes in the collagen bundles. After 4 days, collagen synthesis becomes prominent and the strength of the anastomosis increases^13,19^. After 7 days, the intestinal wall regains the same pressure resistance as the normal intestinal wall^20^. Therefore, we performed a pressure test 5 days later, when collagen proliferation was expected to progress. Consequently, an increase in physical pressure resistance was confirmed, which was considered an important result.

One concern about intraperitoneal application of PGA sheets is that they may form adhesions with surrounding organs^9,21^. In this study, mild adhesions between the intestine and abdominal wall were observed in approximately all rats receiving PGA sheets. However, in the case of double-stapling technique (DST) anastomosis for rectal cancer, since the PGA sheet is used in the narrow pelvic cavity, the small intestine rarely contacts with the anastomotic site; adhesions with other intestinal parts are thought to be rare. Furthermore, no increase in complications associated with the use of PGA sheets was observed in our previous study on the effectiveness of using PGA sheets in combination with DST anastomosis^10^. Although not used for this study, a nano-PGA sheet (NEOVEIL nano^®^; Gunze, Kyoto, Japan) with reduced content of PGA, which is a foreign substance to the body, is expected to decrease adhesions by reducing inflammatory reactions. Further research is needed to determine whether this product could be useful for gastrointestinal anastomosis^9,14^.

This study has limitations. First, this study used rats, which may undergo a different healing process compared to humans. Therefore, there are limits to discussing its effectiveness in the human body. Second, the present tissue evaluation was not performed at the anastomotic site, where fibrotic reactions actually occurred as part of the healing process, but was limited to the normal intestinal wall. Next, owing to the lack of funds for this study and constraints of our experimental facility, the evaluation was performed without anastomosing the intestinal tract or using a stapler, which is often used in clinical surgery.

In conclusion, the PGA sheet formed a fibrotic barrier several days after surgery and contributed to increasing physical pressure resistance by reinforcing the intestinal wall.

## Data Availability

The data analyzed during the current study are available from the corresponding author upon reasonable request.
